# Social support and adolescent normative behavior in the context of rule of law education: the mediating role of educational expectations among Chinese students

**DOI:** 10.3389/fpsyg.2026.1775742

**Published:** 2026-05-21

**Authors:** Bin Guo

**Affiliations:** Faculty of Humanities and Foreign Languages, Xi'an University of Technology, Xi'an, China

**Keywords:** Chinese adolescents, educational expectations, normative behavior, rule of law education, social support

## Abstract

**Background:**

Rule of law education in China emphasizes the cultivation of normative behavior among adolescents, yet the mechanisms through which social support promotes behavioral conformity remain insufficiently understood.

**Objective:**

This study examined the relationships among social support, educational expectations, and normative behavior, investigating whether educational expectations mediate the effects of multi-source social support on adolescent normative behavior within the rule of law education context.

**Methods:**

Data were obtained from the China Education Panel Survey (CEPS), comprising 18,426 seventh and ninth grade students from 112 schools across 28 county-level units in mainland China. Structural equation modeling with bootstrap resampling (5,000 iterations) was employed to test the hypothesized mediation model, and multi-group analyses examined heterogeneity across gender and residential status.

**Results:**

Parental support (β = 0.24, *p* < 0.001), teacher support (β = 0.15, *p* < 0.001), and peer support (β = 0.11, *p* < 0.01) exerted significant positive effects on normative behavior. Educational expectations partially mediated these relationships, with indirect effects ranging from 0.025 to 0.058 (95% CI excluding zero). Teacher support demonstrated significantly stronger effects on educational expectations among rural students (β = 0.24) compared to urban students (β = 0.15, Δχ2 = 6.84, *p* = 0.009).

**Conclusion:**

These results yield associational evidence consistent with a goal-protection framework in which educational aspirations are linked with normative behavior, while pointing to the compensatory role played by teacher support in resource-scarce rural settings. Because the design is cross-sectional, causal conclusions cannot be drawn, yet the observed pattern has practical relevance for supporting adolescent behavioral development within the Chinese rule of law education policy environment.

## 1 Introduction

Adolescent normative behavior promotion is one of the basic objectives of rule of law education in contemporary China. Following the integration of legal education into school education programs, encouraging students‘ submission to social and legal standards has become a pressing interest of educators and policymakers (Dong and Zeb, 2022). Education in the rule of law extends beyond the imparting of legal knowledge. It aims at the all-round development of students' normative consciousness, especially in the area of obedience to rules, compliance with social order, and follow-through on prosocial actions (Shi et al., 2023). Within this educational philosophy, understanding the mechanisms that shape adolescent normative behavior carries profound implications for achieving the goals of citizenship education in Chinese schools (Li, 2024).

The Chinese model of rule of law education (fazhi jiaoyu) sharply contrasts with the Western models of legal socialization. While the Western models usually focus on the importance of rights, adversarial legal thinking, and civic autonomy, fazhi jiaoyu represents a collectivist approach grounded in Confucian values that emphasize harmony, compliance with institutions, and adherence to the officially endorsed behavioral norms (Shi et al., 2023; Dong and Zeb, 2022). This distinction matters here because the behavioral norms studied in this research are based on the official behavioral standards accepted in the Confucian education system.

Social support from family, education institutions, and peer groups is also considered an important element influencing behavioral development in adolescents. Findings have confirmed that the appropriate use of social support networks is very effective in improving behavioral outcomes among adolescents (Bauer et al., 2021). The positive youth development point of view, adolescents who are supported by a variety of sources are likely to develop an inner system of values that are positive and avoid deviant behaviors (Qi et al., 2022). However, the specific pathways through which social support promotes normative behavior, particularly within the rule of law education context, remain insufficiently understood.

This study makes three distinct contributions to the existing literature. First, where previous studies have typically examined how social support shapes adolescent academic achievement or general welfare (Ahmed et al., 2010; Zhang and Qian, 2024), the current research addresses normative behavior under the distinctive institutional framework of fazhi jiaoyu. Second, by combining goal-protection reasoning with expectancy-value theory and the social control perspective, the study develops and empirically tests a mediated model in which educational expectations transmit the effect of multi-source social support onto adolescent normative behavior. Third, while much prior work has treated social capital as a context-free motivational resource, the heterogeneity analysis based on measurement invariance testing identifies teacher support as a compensatory mechanism activated specifically under conditions of rural resource scarcity. The remainder of the paper is organized as follows. Section 2 sets out the theoretical framework and hypotheses. Section 3 describes the data, measures, and analytic strategy. Section 4 reports the empirical results. Section 5 discusses the findings in relation to prior literature and articulates their boundary conditions. Section 6 concludes with implications for the rule of law education policy environment and notes directions for future research.

## 2 Theoretical framework and hypotheses

### 2.1 Normative behavior in the context of rule of law education

Normative behavior in this study refers to the adolescent behavior that is in line with the existing social and legal requirements. This construct can be understood as the absence of problem behaviors like violating rules, aggression, absences, and delinquency, and the presence of behavior in line with the goals of the citizenship education (Abhishek and Balamurugan, 2024). The conceptualization captures the dual thrust of China's rule of law education policy, aiming at reducing behavioral offenses while cultivating positive normative consciousness.

The relevance of normative behavior is grounded in social control theory, which argues that people who have strong attachments to mainstream society are less inclined to participate in criminal activities (Cullen and Wilcox, 2013). Within the Chinese educational setting, normative behavior is a highly significant outcome, signifying the internalization of societal values through moral and legal education. Based on ecological systems theory, the behavior of teenagers is a product of a range of environmental factors, including the individual, family, school, peer, and societal environments (El Zaatari and Maalouf, 2022). The policy framework for rule of law education represents the macro system within which the micro-system variables interact.

### 2.2 Social support as a multi-dimensional construct

According to social support theory, various facets and sources of social support exist and help facilitate adolescent development. On the academic side, the three main sources of social support include parental support, as denoted by emotional nurturance, moral support, and engagement in educational activities; teacher support, which includes academic instruction and behavioral support; and peer support, denoted by friendship and behavioral socialization (Gottlieb, 2017). Research employing latent profile analysis has shown that distinct configurations of support from these multiple sources are differentially associated with adolescent wellbeing and behavioral adjustment (Narainsamy et al., 2024).

The role and value of multiple social support for normative behaviors can be appreciated by understanding social control theories and their application to explaining normative and deviant behaviors. Support from parents increases attachment and provides conventional values; from teachers increases commitment to educational goals; from peers provides normative reference groups (Cullen and Wilcox, 2013). When it comes to rule of law education, these three support providers together comprise the microsystem that socializes students toward ideal behavior patterns. Studies on bioecological models of education have confirmed that combined support from both the family and school environment leads to better developmental outcomes (Tong and An, 2024).

### 2.3 Social support and adolescent normative behavior

Social support and normative behaviors are linked through various mechanisms proposed by different theories. Social elements have been shown to shape behavioral outcomes among juveniles, and social support acts as protective elements against juvenile delinquency and antisocial behaviors (Abhishek and Balamurugan, 2024). Building on the attachment-commitment-belief bonds outlined above, empirical work has consistently linked consistent multi-source support to reductions in norm-violating behaviors (Cullen and Wilcox, 2013).

Empirical studies have found the positive impact of diverse sources of support on behavior among adolescents. Research conducted on teacher support revealed a correlation between teacher support and a decrease in externalizing problem behaviors among Chinese youth (Zhu et al., 2025). Research related to peer relationship studies proved peer attachment affected prosocial behavior and reduced conduct problems (Schoeps et al., 2020). Under the rubric of rule of law education, social supports that come from multiple sources help to reinforce the norms specified by educational policy, establishing a socialization context that leads to conformity. Based on the theoretical and empirical foundations:

H1: Social support (parental, teacher, peer) has a significant positive effect on adolescent normative behavior.

### 2.4 Social support and educational expectations

Educational expectations can be defined as the beliefs that adolescents and others, such as parents, hold over future educational attainment. There is meta-analytic evidence that suggests that the effects of parents' educational expectations affect academic motivation and developmental outcomes (Pinquart and Ebeling, 2020). Aspirations can be influenced by supportive relationships in numerous ways, such as through warm parenting practices, which indicate high educational aspirations for one's offspring, teacher support, which encourages individuals to believe in their academic possibilities, and peers who emphasize academic success.

Studies have shown that a match between parental and teenage academic expectations predicts positive relationship quality and levels of engagement (Song et al., 2024). Academic socialization processes mediated by nurturing parent-adolescent relationships prove to be important predictors for students‘ belief systems (Woreta, 2024). Studies focusing on parental involvement have found positive correlations between perceived expectations of parents and students' aspirations (Wang and Tambi, 2024). Within the rule of law education context, educational expectations represent internalized goals that connect current behavioral choices to future outcomes. Thus:

H2: Social support has a significant positive effect on educational expectations.

The theoretical model here postulates a one-directional association running from social support to educational expectations. This directionality matches the developmental order implied by the expectancy-value tradition, in which supportive ties contribute to the formation of achievement attitudes (Eccles and Wigfield, 2020), though the assumption may be somewhat oversimplified. Adolescents who already hold strong expectations can actively seek or attract more support from their parents and educators, a pattern that implies possible reverse causation. Given the cross-sectional nature of the current study, these competing pathways cannot be fully disentangled. The one-directional specification is retained on grounds of theoretical parsimony and on evidence from longitudinal work suggesting that social support tends to precede shifts in motivational states (Ahmed et al., 2010; Zhang and Qian, 2024). Adjudication between the competing directional accounts ultimately requires longitudinal or experimental designs beyond the scope of the present analysis.

### 2.5 Educational expectations and normative behavior

Expectancy-value theory is the theoretical base for examining how education-related expectations influence normative behavior. Expectancy-Value Theory holds that people's motivation and behavior are guided by their beliefs of success and personal value associated with achieving particular goals (Eccles and Wigfield, 2020). When adolescents hold high expectations for educational attainment, they develop stronger commitment to behaviors that support academic success and protect their future opportunities.

The connection between educational expectations and normative behavior takes place through a goal-protection process that is specifically relevant in a rule of law context for education. Adolescents who hold elevated educational aspirations recognize that norm-violating behaviors such as rule-breaking, truancy, fighting, or cheating jeopardize their academic futures and social standing. Empirical support shows that autonomy support in parenting interacts in academic motivation through the fulfillment of psychological needs, while achievement goal orientation mediates the relationship between parenting and educational outcomes (Çelik, 2024; Xu et al., 2024). These results make clear the fact that the presence of educational expectations exerts regulatory functions in terms of facilitating social and institutional compliance. The teenagers possessing high educational expectations feel the need to behave in a manner consistent with the behavioral criteria required in order to regulate their educational path. Therefore:

H3: Educational expectations have a significant positive effect on adolescent normative behavior.

### 2.6 The mediating role of educational expectations

Based on the above-mentioned theory, the current research suggests that educational expectation mediates the effect of multi-source social support on normative behavior. It is expected that multi-source social support increases adolescents' educational expectation (H2); higher educational expectation activates goal-protection motivation leading to normative behavior (H3). Thus, the effect of social support on normative behavior can be explained not only by the direct effect through enhanced social ties but also by the indirect effect of raising educational expectation.

Empirical evidence regarding this mediation model relies upon research validating that social support impacts behavior outcomes through sequential mediation involving motivational beliefs (Zhang and Qian, 2024). The role of academic motivation, a proposed mediator, between social support and desired behavior outcomes has been validated within the adolescent demographic (Ahmed et al., 2010). Some findings are from meta-analyses that have confirmed links for social support and prosocial behavioral outcomes (Wang et al., 2024), while research specifically examining Chinese adolescents that has revealed mediating factors that separate teacher support from decreased problem behaviors (Zhu et al., 2025).

The mediation model proposed integrates concepts from social support theories, social control theories, ecological system theories, and expectancy-value theories. A theoretical integration suggests that social support theory defines the determinants of support, sources, and dimensions, while social control theory defines the manner by which support builds the ties that prevent deviant behavior, ecological systems theory contextualizes this process within the rule of law education macrosystem, and expectancy-value theory defines the cognitive and motivational process by which expectations affect behavior. Based on this theoretical integration:

H4: Educational expectations mediate the relationship between social support and adolescent normative behavior.

To clarify how the theoretical framework maps to the empirical operationalization of this study, social support theory forms the theoretical basis for the three sources from which social support is derived, operationalized as three latent constructs: parental support (five-item scale), teacher support (four-item scale), and peer support (four-item scale), on a four-point Likert scale; social control theory provides the theoretical foundation for the dependent variable, normative behavior, operationalized as a problem behavior scale (10-item reverse coded) where high scores reflect better normative behavior; ecological systems theory explains the context in which the multi-sourced support framework operates (i.e., the rule of law education macrosystem) and explains the motivation for urban and rural comparison groups; while expectancy-value theory underpins the mediating variable, educational expectations, operationalized as a two-indicator latent variable covering self and parental expectations on an eight-point ordinal scale.

## 3 Materials and methods

### 3.1 Data source and participants

In this study, we use data collected in the China Education Panel Survey (CEPS) conducted by the National Survey Research Center of Renmin University of China. The CEPS had a nationally representative longitudinal design and was carried out in the 2013–2014 academic year. The CEPS has a stratified multistage sample design based on probability proportion to size sampling. The sampling frame consisted of 28 county units, with 112 schools and 438 classrooms sampled, covering major and minority regions in eastern, central, and western China.

Representative groups for the study were seventh-grade students, marking the first critical developmental phase where basic norms of behavior are shaped, and ninth-grade students, which represent the most formative phase of adolescence where seeds for future learning are sown. Sampling for the study involved the use of questionnaires, where 20,000 students completed the questionnaire independently. Cases that had missing data on the variables of study were deleted, leaving a sample of 18,426 students.

The dataset collection for CEPS was approved by the Institutional Review Board at the Renmin University of China. Informed consent was sought from all the students participating in the study and their guardians. The current study is a secondary analysis because the dataset is anonymous and publicly available through the official CEPS repository.

Sampling for the CEPS employed a stratified, multi-stage probability proportional to size (PPS) sampling method. The first stage involved selecting 28 county-level samples representing eastern, central, and western parts of China, as well as urban and rural regions. The second stage involved random selection of 112 schools in the selected counties, while the third stage involved random selection of 438 classrooms in the selected schools. Such sampling ensures geographic and socioeconomic diversity, thus leading to strong representation of junior secondary students in mainland China. Potential sources of sampling bias included non-response at the school level and the exclusion of out-of-school youth. Thus, the findings are generalizable only to junior secondary students enrolled in schools. Missing values of independent variables were managed through listwise deletion. Chi-square tests were used to determine if there were any differences between the retained sample (*N* = 18,426) and the complete survey sample (*N* approximately 20,000) in terms of gender, grade, and hukou status. No statistically significant differences were found (all *p* > 0.10).

### 3.2 Measures

#### 3.2.1 Independent variable: social support

Social support was treated as a multi-dimensional construct comprising three sources consistent with ecological systems theory. Parental support was assessed with five items covering emotional support (e.g., “My parents encourage me when I face difficulties”), academic involvement (e.g., “My parents frequently ask about how things are going at school”), and communication (e.g., “I can freely discuss my concerns with my parents”). Teacher support was assessed with four items tapping teacher-student relationship quality and academic encouragement (e.g., “My teachers pay attention to my class performance,” “My teachers praise my efforts when I make progress,” and “My teachers are willing to help me whenever I have questions”). Peer support was assessed with four items reflecting peer relationship quality and the prosocial classroom environment (e.g., “I get along well with my classmates,” “My classmates are willing to help me when I need it,” and “I feel accepted by my peers at school”). All items used a four-point Likert scale (1 = completely disagree to 4 = completely agree), with higher scores indicating greater perceived support.

#### 3.2.2 Mediating variable: educational expectations

Expectations about education were measured through two indicators that were based on the framework of expectancy-value theory, tapping both self-expectations and perceived parental expectations. The two questions asked were “What level of education do you expect to achieve?” and “What level of education do you think that your parents expect you to achieve?” Response categories were measured ordinally as follows: (1) drop out now, (2) junior high school, (3) vocational secondary school, (4) vocational high school, (5) regular high school, (6) junior college, (7) bachelor's degree, and (8) graduate degree or above. The two indicators were used to create a latent variable for educational expectations.

It is more beneficial to consider them as reflective indicators of one latent variable rather than computing a mean of the two variables because it takes into consideration the different reliabilities of the two items, in accordance with good practice in latent variable modeling (Eccles and Wigfield, 2020). The two items are significantly correlated (*r* = 0.52, *p* < 0.001), thus they can represent one latent expectation. This dual indicator measure can capture both individualized aspirations and externally pressured expectations.

#### 3.2.3 Dependent variable: normative behavior

Normative behavior was operationalized as the adolescents‘ behavior that adhered to legitimate social norms and expectations, in keeping with the rule of law educational goals. The indicator consisted of a ten-item problem behavior scale designed for assessing behavioral offenses in the previous semester: (1) being late for school, (2) being absent without permission, (3) cheating on examinations, (4) using profane language, (5) physical fighting, (6) bullying classmates, (7) damaging public or others' property, (8) smoking, (9) drinking alcohol, and (10) visiting internet cafés. Students indicated frequency on a five-point Likert scale (1 = never to 5 = always). Items were reverse-coded so that higher scores indicate greater normative behavior, directly reflecting the rule of law education goal of reducing behavioral violations while promoting rule compliance.

The rationale for this operationalization is underpinned by the social control theory, which suggests that normative behavior should be best conceptualized through the non-occurrence or suppression of deviance (Cullen and Wilcox, 2013). This choice is also consistent with the use of problem behavior scales based on the CEPS among previous Chinese adolescent research studies, and these scales measured behavioral conformity (Shi et al., 2023). The drawback of this measurement strategy is that it focuses on one aspect of normative behavior, which is violation, without considering its proactive and prosocial aspects. Future research can explore using additional scales to measure normative behaviors as an outcome variable.

#### 3.2.4 Control variables

Covariates included gender (male = 1, female = 0), age, grade level (7th = 0, 9th = 1), hukou status (urban = 1, rural = 0), only-child status (yes = 1, no = 0), family socioeconomic status (composite measure of parental occupation and household assets), and highest parental education level.

### 3.3 Statistical analysis

In intuitive terms, the analytic strategy proceeds in three conceptual steps. First, a measurement phase verifies that the survey items reliably tap the five latent constructs (parental, teacher, and peer support; educational expectations; normative behavior) through reliability, convergent, and discriminant validity checks. Second, a structural phase estimates how these five constructs are linked in a single SEM, yielding both the direct association of each support source with normative behavior and the indirect pathway running through educational expectations. Third, a heterogeneity phase re-estimates the structural phase separately for gender and urban-rural subgroups, with measurement invariance established first so that group differences reflect substantive rather than instrumental variation.

**Figure 1:**
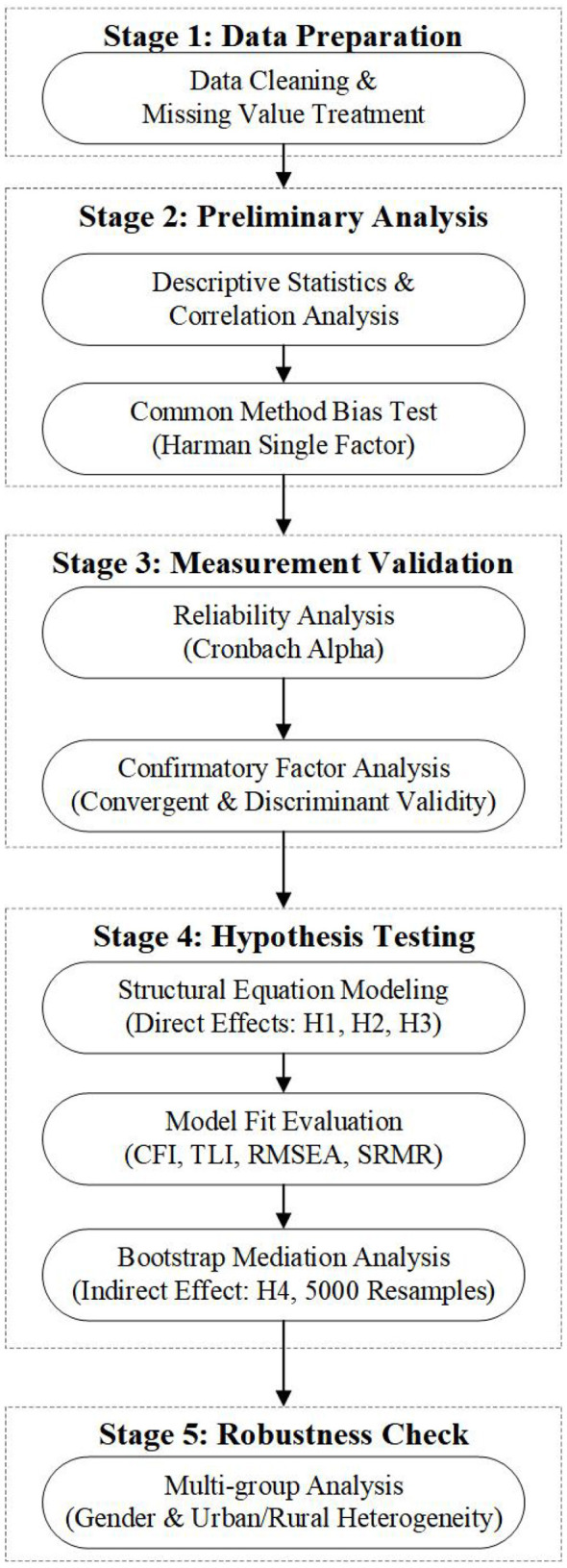
Analytical procedure for structural equation modeling.

Data analysis proceeded through a systematic five-stage procedure using SPSS 26.0 for preliminary analyses and AMOS 24.0 for structural equation modeling, as illustrated in Figure 1.

The analysis process started by preparing the data, which involved data cleaning and the treatment of missing values. The initial analysis included the use of descriptive statistics, correlation analysis, and an examination for common method bias by employing Harman's single-factor test, where appropriate levels were determined by the variance explained by a single factor, which was below 50%. Measurement validation included reliability analysis using Cronbach's alpha coefficients (threshold > 0.70) and confirmatory factor analysis. Convergent validity was assessed through standardized factor loadings (> 0.50) and average variance extracted (AVE > 0.50). Discriminant validity was evaluated using the Fornell-Larcker criterion.

Hypothesis testing employed structural equation modeling to examine direct effects (H1, H2, H3) and mediation effects (H4). Model fit was evaluated using multiple indices: χ^2^/df < 3.0, CFI > 0.90, TLI > 0.90, RMSEA < 0.08, and SRMR < 0.08. The mediation effect of educational expectations was tested using bootstrap resampling with 5,000 iterations to generate bias-corrected 95% confidence intervals, where intervals excluding zero indicate significant mediation. Multi-group analyses examined potential heterogeneity across gender and urban/rural residential status after establishing measurement invariance.

Because CEPS provides a single wave of cross-sectional, observational data, the analytic strategy faces identification boundaries that should be stated plainly. Students with stronger intrinsic motivation or higher conscientiousness may simultaneously seek more social support, report higher educational aspirations, and display more normative behavior, which could inflate the estimated coefficients even after conditioning on the covariate set (gender, age, grade, hukou, only-child status, family socioeconomic status, and highest parental education). The single-wave structure of the data also precludes identification strategies that would otherwise mitigate such concerns, including instrumental variables without credible exclusion restrictions, individual fixed-effects models that require repeated measures, and propensity-score designs that need a well-defined treatment. We therefore treat the SEM estimates as associations conditional on observed covariates rather than as causal effects, and we flag longitudinal follow-up with multi-wave CEPS rounds as the appropriate route to formal robustness testing and causal identification.

## 4 Results

### 4.1 Descriptive statistics

The total sample used in the analyses was 18,426, after the removal of cases where there was missing information on the relevant study variables. Table 1 above shows the demographic information of the respondents. The sample used was fairly representative of the gender distribution of the respondents, where 51.2% (*n* = 9,433) were male, and 48.8% (*n* = 8,993) were female. With respect to the grade levels, 52.4% (*n* = 9,653) of the respondents were in grade seven, while 47.6% (*n* = 8,773) were in grade nine. The prevalence of household registration status was revealed to be 42.8% (*n* = 7,886) for those with urban hukou and 57.2% (*n* = 10,540) with rural hukou, representing the prevalence among Chinese secondary school students. Concerning family structure, 47.3% (*n* = 8,715) participants were only children, and 52.7% (*n* = 9,711) had brothers or sisters. The mean age of participants was 14.23 years (SD = 1.38), with participants aged between 12 to 17 years. As for parental education levels, most fathers (34.6%) and mothers (36.2%) finished their education at the level of junior high school education; next was senior high school or vocational school completion (fathers: 28.4%; mothers: 25.7%). The characteristics of the samples show there is adequate representation in terms of demographics considered for the evaluation of social support, educational expectations, and social norms for Chinese adolescents participating in the rule of law education. The representative distribution in the samples in terms of gender and grade levels, and good inclusion of both urban and rural students, enhances the generalizability of the findings to the Chinese junior secondary school-going students.

**Table 1:** Demographic characteristics of participants (*N* = 18,426).

Variable	Category	*n*	%
Gender	Male	9,433	51.2
	Female	8,993	48.8
Grade level	7th grade	9,653	52.4
	9th grade	8,773	47.6
Hukou status	Urban	7,886	42.8
	Rural	10,540	57.2
Only-child status	Yes	8,715	47.3
	No	9,711	52.7
Father's education	Primary school or below	2,578	14.0
	Junior high school	6,375	34.6
	Senior high school/Vocational	5,233	28.4
	Junior college	2,026	11.0
	Bachelor's degree or above	2,214	12.0
Mother's education	Primary school or below	3,317	18.0
	Junior high school	6,672	36.2
	Senior high school/Vocational	4,735	25.7
	Junior college	1,953	10.6
	Bachelor's degree or above	1,749	9.5
Age (years)	M = 14.23, SD = 1.38, Range: 12–17		

### 4.2 Preliminary analyses

Before engaging in hypothesis testing, some initial analyses were carried out to determine the nature of the distribution for the variables of interest and examine bivariate relationships. Table 2 presents the descriptive statistics and correlation matrix for each primary variable of the study. Results from descriptive statistics reveal that moderate to high levels of perceived social support exist, where support from parents (M = 3.21, SD = 0.64), support from teachers (M = 2.89, SD = 0.71), and support from peers (M = 3.15, SD = 0.58) each scored higher than the scale median. Expectations of education were high in the participants as their levels were found near the upper end of the scale, which is an eight-point scale (M = 6.50, SD = 1.26). Normative behavior was found to be low as the mean score on the reverse-scored scale was 4.36 (SD = 0.52) indicating that the subjects reported low levels of problem behaviors.

**Table 2:** Descriptive statistics and correlation matrix of main study variables (*N* = 18,426).

Variable	M	SD	1	2	3	4	5
1. Parental support	3.21	0.64	—				
2. Teacher support	2.89	0.71	0.46^***^	—			
3. Peer support	3.15	0.58	0.39^***^	0.44^***^	—		
4. Educational expectations	6.50	1.26	0.44^***^	0.35^***^	0.30^***^	—	
5. Normative behavior	4.36	0.52	0.38^***^	0.32^***^	0.28^***^	0.35^***^	—

^***^p < 0.001. Parental Support, Teacher Support, and Peer Support measured on 4-point scales (1–4); Educational Expectations is shown here as the average of the two indicators (self-expectations and parental expectations) for interpretability of descriptive statistics, while the SEM treats the two items as reflective indicators of a single latent construct (see Measures), measured on an 8-point ordinal scale (1–8); Normative Behavior measured on 5-point scale (1–5, reverse-coded, higher scores indicate greater normative behavior).

**Figure 2:**
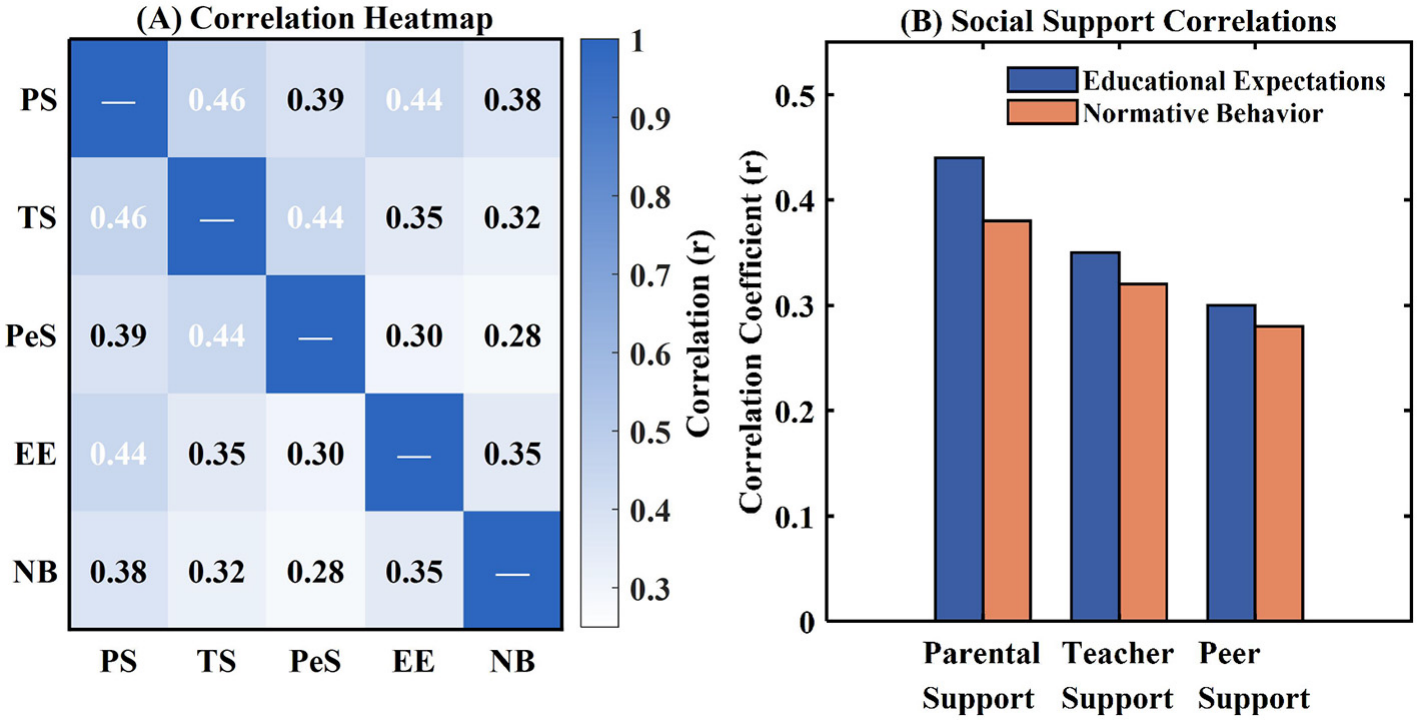
Correlation patterns among study variables. **(A)** Correlation heatmap matrix showing pairwise Pearson correlations among parental support (PS), teacher support (TS), peer support (PeS), educational expectations (EE), and normative behavior (NB), in which darker blue shading indicates stronger positive correlation. **(B)** Grouped bar chart comparing the correlations of parental, teacher, and peer support with educational expectations (blue bars) and normative behavior (orange bars).

Pearson correlation results showed that all forms of social support were significantly and positively related to educational expectations and normative behavior, providing initial evidence to confirm the hypotheses. As illustrated in Figure 2, parental social support had the highest correlation with educational expectations (*r* = 0.44, *p* < 0.001) and normative behavior (*r* = 0.38, *p* < 0.001), followed by teacher social support (*r* = 0.35 and *r* = 0.32 respectively) and peer social support (*r* = 0.30 and *r* = 0.28 respectively). The correlation relationship for the expectation of education to the normative manner was both significant and positive (*r* = 0.35, *p* < 0.001), by supporting the hypothesized path. The value for the variance inflation factors ranged from 1.12 to 1.67, below the cut-off value of 10, ensuring that there were no problems due to multicollinearity. In checking for common method bias, Harman's single-factor test revealed that the first unrotated factor only explained 28.6% of the total variance, which is far below the 50% cut-off level. These results show that there are no problems associated with common method bias.

### 4.3 Measurement model

Before proceeding with the examination of the structural model, there was a need for a confirmatory factor analysis, which helped analyze the measurement model and its psychometric properties. The measurement model had five latent variables, which were support from parents, support from teachers, support from peers, educational expectations, and normative behavior. As presented in Table 3, the model demonstrated acceptable fit to the data, with χ^2^/df = 2.87, CFI = 0.952, TLI = 0.944, RMSEA = 0.042, and SRMR = 0.038, all meeting the established criteria for good model fit. Analysis of reliability showed that all constructs had satisfactory internal consistency, as supported by Cronbach's alpha values that ranged between 0.78 and 0.89, surpassing the minimum acceptable standard of 0.70. Values for the composite reliability ranged from 0.80 to 0.90, further establishing the reliability of the scales used.

**Table 3:** Measurement model: reliability and validity assessment.

Construct	Items	α	CR	AVE	$\sqrt{AVE}$	1	2	3	4	5
1. Parental support	5	0.85	0.86	0.55	0.74	—				
2. Teacher support	4	0.82	0.83	0.55	0.74	0.46	—			
3. Peer support	4	0.78	0.80	0.52	0.72	0.39	0.44	—		
4. Educational expectations	2	0.81	0.82	0.67	0.82	0.44	0.35	0.30	—	
5. Normative behavior	10	0.89	0.90	0.53	0.73	0.38	0.32	0.28	0.35	—

α, Cronbach's alpha; CR, composite reliability; AVE, average variance extracted; $\sqrt{AVE}$ = square root of AVE (shown in diagonal for discriminant validity assessment). Off-diagonal values represent inter-construct correlations. Model fit: χ2/df = 2.87, CFI = 0.952, TLI = 0.944, RMSEA = 0.042, SRMR = 0.038.

**Figure 3:**
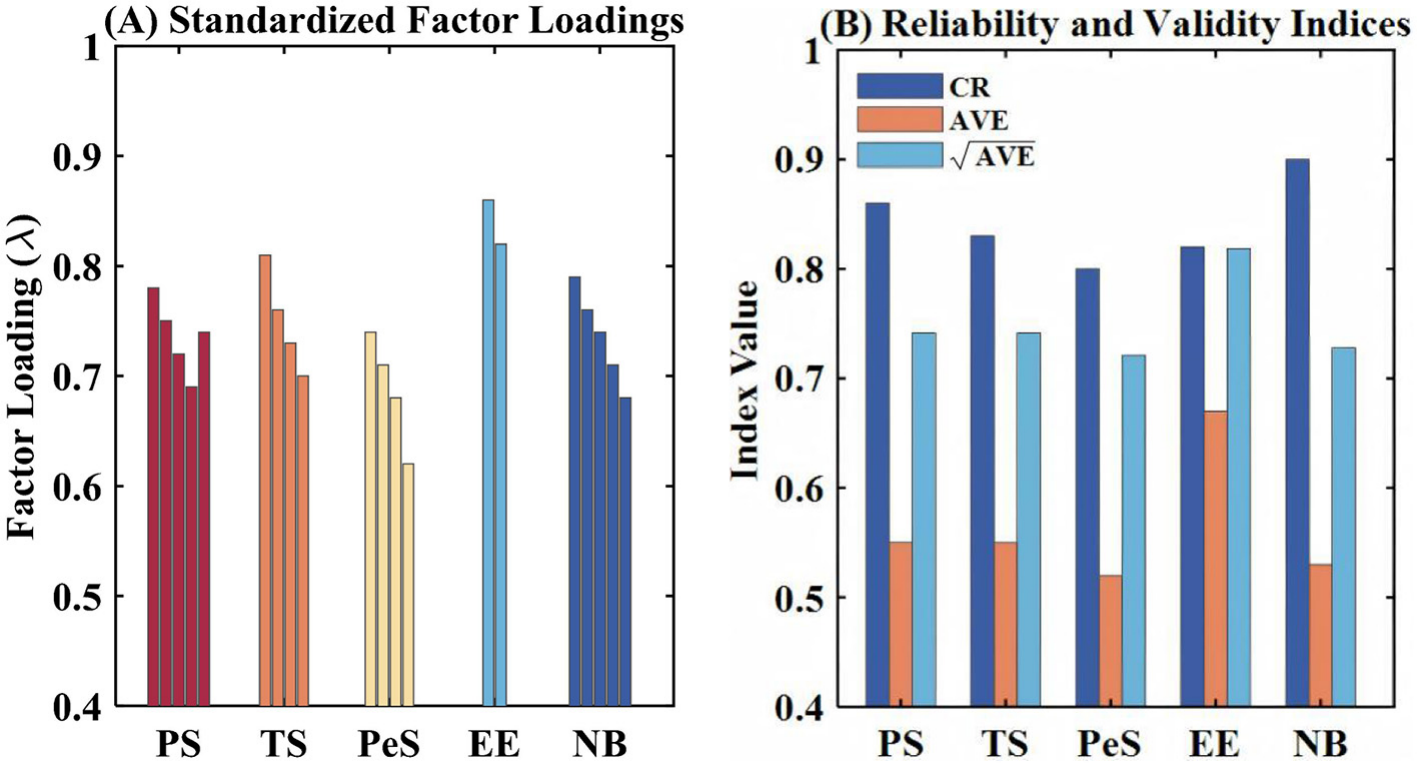
Measurement model validation. **(A)** Standardized factor loadings of the indicators for the five latent constructs (PS, TS, PeS, EE, NB). **(B)** Reliability and validity indices for each construct, showing composite reliability (CR, dark color), average variance extracted (AVE, medium color), and the square root of AVE (√AVE, light color).

The results of the convergent validity are calculated utilizing factor loadings and average variance extracted. The factor loadings of all constructs are significant and above the threshold of 0.50, which ranges from 0.62 to 0.86 (Figure 3). This verifies that the latent construct was adequately measured by the indicators. The average variance extracted of all constructs was above the threshold of 0.50, which ranges from 0.52 to 0.67. Discriminant validity was tested using the Fornell-Larcker criterion, where the criteria require that the square root of AVE values for all constructs should exceed the correlation values between constructs. This shows that there is satisfactory discriminant validity since the square root of AVE values for all constructs exceeded their correlation values. This confirms that there is satisfactory reliability, validity, and discriminant validity of the measurement model, which is a solid foundation for a subsequent structural model test on hypotheses about relationships between social support, expectations of education outcomes from rule of law education, and normative behavior.

### 4.4 Structural model and hypothesis testing

The structural equation model was estimated to test the hypothesized relationships among social support, educational expectations, and normative behavior. As presented in Table 4, the structural model demonstrated good fit to the data, with χ2/df = 2.93, CFI = 0.948, TLI = 0.941, RMSEA = 0.045, and SRMR = 0.042, all meeting the established criteria for acceptable model fit. The results provided support for the hypothesized direct effects. Regarding H1, all three forms of social support were found to have significant positive relationships to normative behavior: parental support (β = 0.24, *p* < 0.001), teacher support (β = 0.15, *p* < 0.001), and peer support (β = 0.11, *p* < 0.01) all played important parts. It appears that those youths perceiving greater amounts of parental, teacher, and peer support will have higher probabilities of adhering to normative forms of behavior as it applies to rule of law schooling.

**Table 4:** Structural model results: direct effects and mediation analysis (*N* = 18,426).

Path	β	SE	*t*	*p*	Result
Direct effects on normative behavior					
Parental support → Normative behavior	0.24	0.02	12.00	< 0.001	H1a supported
Teacher support → Normative behavior	0.15	0.02	7.50	< 0.001	H1b supported
Peer support → Normative behavior	0.11	0.02	5.50	< 0.01	H1c supported
Educational expectations → Normative behavior	0.18	0.02	9.00	< 0.001	H3 supported
Direct effects on educational expectations					
Parental support → Educational expectations	0.32	0.02	16.00	< 0.001	H2a supported
Teacher support → Educational expectations	0.19	0.02	9.50	< 0.001	H2b supported
Peer support → Educational expectations	0.14	0.02	7.00	< 0.001	H2c supported
Indirect effects (Bootstrap 95% CI)	Effect	Boot SE	LL CI	UL CI	
Parental support → EE → Normative behavior	0.058	0.006	0.046	0.071	H4a supported
Teacher support → EE → Normative behavior	0.034	0.005	0.025	0.045	H4b supported
Peer support → EE → Normative behavior	0.025	0.005	0.016	0.036	H4c supported

β, standardized coefficient; SE, standard error; LL CI, lower limit of 95% confidence interval; UL CI, upper limit of 95% confidence interval; EE, Educational Expectations. Model fit: χ2/df = 2.93, CFI = 0.948, TLI = 0.941, RMSEA = 0.045, SRMR = 0.042. Bootstrap resampling with 5,000 iterations was used to generate bias-corrected 95% confidence intervals for indirect effects; intervals excluding zero indicate significant mediation. Direct and indirect effects are reported as standardized coefficients from a single SEM that specifies parental, teacher, and peer support as exogenous latent predictors, educational expectations as a mediating latent variable, and normative behavior as the latent outcome. All models controlled for gender, age, grade, hukou, only-child status, family socioeconomic status, and highest parental education.

**Figure 4:**
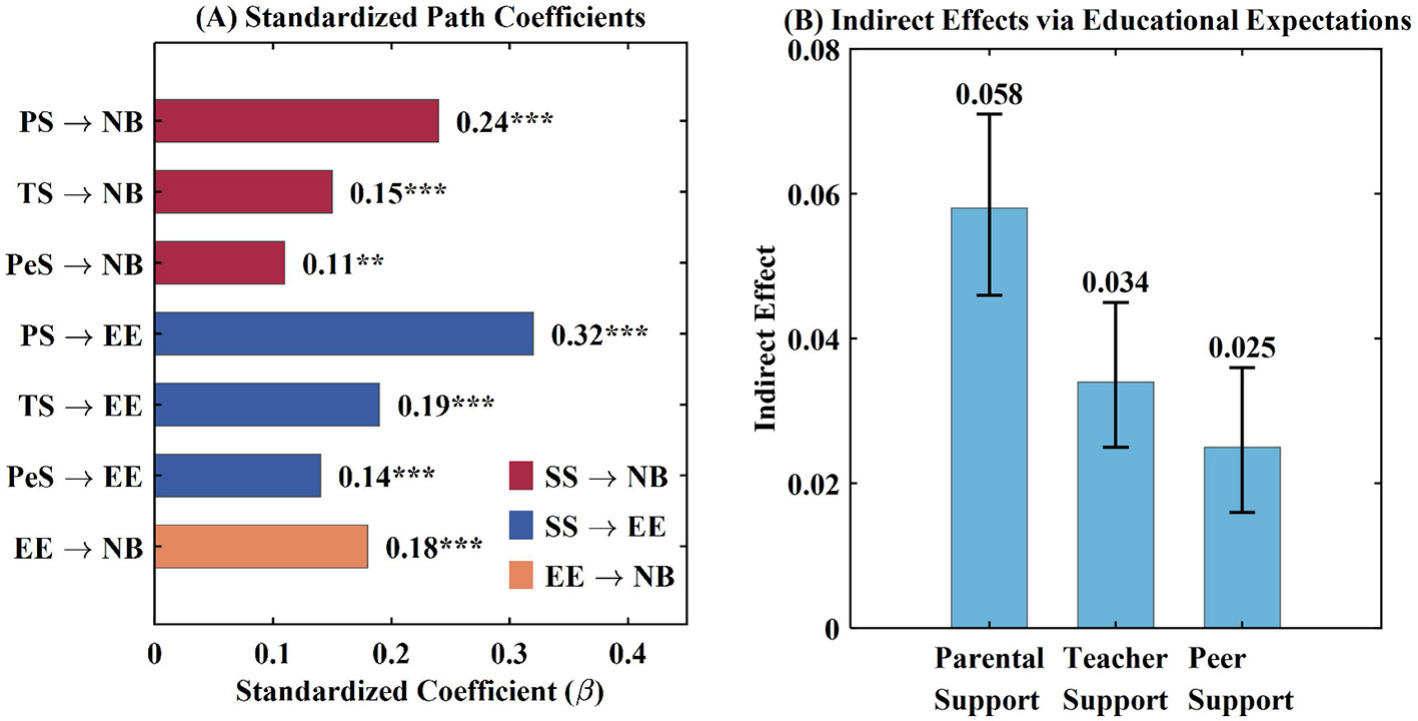
Structural model results. **(A)** Standardized path coefficients (β) showing direct effects of three social support sources (parental support PS, teacher support TS, peer support PeS) on normative behavior (NB) and educational expectations (EE), as well as the effect of educational expectations on normative behavior. **(B)** Indirect effects of parental, teacher, and peer support on normative behavior via educational expectations, estimated using bootstrap resampling with 5,000 iterations, in which error bars represent 95% bias-corrected confidence intervals. **p < 0.01; ***p < 0.001.

The findings also supported that social support positively impacts educational expectations (H2) after controlling for all variables. It was found that the strongest relationship of social support is demonstrated by parental support (β = 0.32, *p* < 0.001), followed by teacher support (β = 0.19, *p* < 0.001) and peer support (β = 0.14, *p* < 0.001). Educational expectations, in turn, demonstrated a significant positive effect on normative behavior (β = 0.18, *p* < 0.001), supporting H3. As illustrated in Figure 4, the mediation analysis using bootstrap resampling with 5,000 iterations revealed significant indirect effects of all social support dimensions on normative behavior through educational expectations: parental support (indirect effect = 0.058, 95% CI [0.046, 0.071]), teacher support (indirect effect = 0.034, 95% CI [0.025, 0.045]), and peer support (indirect effect = 0.025, 95% CI [0.016, 0.036]). The confidence intervals excluding zero confirmed significant mediation effects, providing support for H4. The percentage of the total effect that was mediated by education expectations ranged from 18.5% to 19.5% for the three dimensions of social support, pointing toward partial mediation whereby social support impacts normative behavior both directly through strengthened social bonds, and indirectly through strengthened education expectations.

For a better understanding of the size of the effects, the standardized direct path coefficients denote the small to medium effect sizes (with a beta of approximately 0.10 denoting a small effect size, and 0.30 denoting a medium effect size). The parental support effect (beta of 0.24) represents an effect of practical importance, whereas the teacher and peer support effects reflect smaller effect sizes. The total effects were as follows: parental support effect of 0.298, teacher support effect of 0.184, and peer support effect of 0.135. The percentage of the indirect effect sizes with respect to the total effects was: 19.5%, 18.5%, and 18.5%, respectively. Within the SEM framework, the standardized β coefficients reported here and the SD-unit predicted increases presented in the urban-rural comparison below are themselves standardized effect size metrics, belonging to the Cohen's d family and directly interpretable using conventional small (0.10), medium (0.30), and large (0.50) benchmarks (Kline, 2023).

### 4.5 Supplementary analyses

To examine the robustness of results and possible differences between subgroups, multi-group analyses were conducted according to gender and residential status. Prior to comparing structural path coefficients, equality of measurements was confirmed by a sequential test. The configural invariance model demonstrated acceptable fit (CFI = 0.945, RMSEA = 0.048), and the metric invariance model showed no significant deterioration in fit (ΔCFI = 0.003), confirming that the factor loadings were equivalent across groups and enabling meaningful comparison of structural relationships.

As shown in Table 5, the results of the multiple group analysis by gender revealed that the structural model fitted both males and females, as all the proposed paths were significant in both groups. Parental support had a marginally stronger positive association with normative behavior for female students (β = 0.27, *p* < 0.001) than for male students (β = 0.21, *p* < 0.001). However, the difference was found to be non-significant using the chi-square difference test (Δχ2 = 2.84, *p* = 0.092). This indicates that the mediating role of expectations regarding education is equivalent for both males and females, implying that the process of goal protection works equivalently for males and females in the domain of education for rule of law.

**Table 5:** Multi-group analysis results by gender (*N* = 18,426).

Path	Male (*n* = 9,433)	Female (*n* = 8,993)	*Δχ*2	*p*
Direct effects	β	β		
Parental support → Normative behavior	0.21^***^	0.27^***^	2.84	0.092
Teacher support → Normative behavior	0.14^***^	0.16^***^	0.42	0.516
Peer support → Normative behavior	0.13^**^	0.09^*^	1.26	0.262
Parental support → Educational expectations	0.30^***^	0.34^***^	1.15	0.284
Teacher support → Educational expectations	0.18^***^	0.20^***^	0.38	0.538
Peer support → Educational expectations	0.12^***^	0.16^***^	1.64	0.200
Educational expectations → Normative behavior	0.16^***^	0.20^***^	1.42	0.233
Indirect effects	Effect	Effect		
Parental support → EE → NB	0.048	0.068	—	—
Teacher support → EE → NB	0.029	0.040	—	—
Peer support → EE → NB	0.019	0.032	—	—

^*^p < 0.05. ^**^p < 0.01. ^***^p < 0.001. EE, Educational Expectations; NB, Normative Behavior. No statistically significant group differences were found between male and female students. Multi-group SEM with factor loadings constrained to equality (metric invariance: ΔCFI = 0.003). Standardized beta reported per group. Delta-chi-square tests compare constrained vs. unconstrained models.

As presented in Table 6, the analysis comparing students with urban and rural hukou revealed notable differences. Parental support demonstrated a significantly stronger effect on both educational expectations (β = 0.36 vs. 0.28, Δχ2 = 5.23, *p* = 0.022) and normative behavior (β = 0.28 vs. 0.21, Δχ2 = 4.12, *p* = 0.042) among urban students compared to rural students. By contrast, teacher support had a strongly positive and significantly greater effect on expectations regarding education for rural youths (β = 0.24, *p* < 0.001) than for urban youths (β = 0.15, *p* < 0.001), with a statistical difference (Δχ2 = 6.84, *p* = 0.009). This result shows that the teachers play a significantly compensatory role in influencing the educational aspirations of the adolescents in the rural areas, where the educational resources in the families are relatively limited. The value of the indirect effect of teacher support on educational expectations was higher for the rural students (indirect effect = 0.046) in contrast to the urban students (indirect effect = 0.026), and this indicates the compensatory effect of teacher support.

**Table 6:** Multi-group analysis results by residential status (*N* = 18,426).

Path	Urban (*n* = 7,886)	Rural (*n* = 10,540)	*Δχ*2	*p*
Direct effects	β	β		
Parental support → Normative behavior	0.28^***^	0.21^***^	4.12	0.042^*^
Teacher support → Normative behavior	0.12^***^	0.18^***^	3.56	0.059
Peer support → Normative behavior	0.09^**^	0.13^**^	1.48	0.224
Parental support → Educational expectations	0.36^***^	0.28^***^	5.23	0.022^*^
Teacher support → Educational expectations	0.15^***^	0.24^***^	6.84	0.009^**^
Peer support → Educational expectations	0.11^**^	0.17^***^	3.42	0.064
Educational expectations → Normative behavior	0.17^***^	0.19^***^	0.45	0.502
Indirect effects	Effect	Effect		
Parental support → EE → NB	0.061	0.053	—	—
Teacher support → EE → NB	0.026	0.046	—	—
Peer support → EE → NB	0.019	0.032	—	—

^*^p < 0.05. ^**^p < 0.01. ^***^p < 0.001. EE, Educational Expectations; NB, Normative Behavior. Bold paths indicate statistically significant group differences. Measurement invariance established: Configural model CFI = 0.945, RMSEA = 0.048; Metric invariance ΔCFI = 0.003. Multi-group SEM with factor loadings constrained to equality. Standardized beta reported per group. Delta-chi-square tests compare constrained vs. unconstrained models.

With respect to the size of the effect, a unit increase in perceived teacher support was estimated to predict a 60% larger increase in educational aspirations for rural youth compared to their urban settings. Predicted increases in education expectations due to a one SD increase in teacher support are as follows: rural youth = an increase of 0.30 (95% confidence interval [CI] 0.22, 0.38); urban youth = an increase of 0.19 (95% CI 0.13, 0.25); difference = 0.11 (95% CI 0.02, 0.20), *p* = 0.018. These results can be considered meaningful because there are clear ceiling effects in urban youth expectations (M = 6.83 vs. 6.24 for rural youth).

To gauge how vulnerable the main associations are to unmeasured confounding, the E-value approach proposed by VanderWeele and Ding (2017) was applied to the four principal direct paths in the structural model. An E-value translates a standardized coefficient into the minimum strength of association, on the risk-ratio scale, that an unobserved confounder would need to have with both the predictor and the outcome, beyond the measured covariates, to reduce the estimated path to the null. Using the standard continuous-outcome conversion RR = exp(0.91 × β), the parental support → normative behavior path yielded an E-value of 1.80 for the point estimate and 1.69 for the lower 95% CI bound; the teacher support path yielded 1.56 and 1.45; the peer support path yielded 1.45 and 1.33; and the educational expectations → normative behavior path yielded 1.64 and 1.53. An unmeasured confounder would therefore need to associate with both sides of each relationship at risk-ratio magnitudes of at least 1.33 to 1.69 (CI bound) to explain away the observed associations after conditioning on gender, age, grade, hukou, only-child status, family socioeconomic status, and highest parental education. These magnitudes exceed the measured associations of most demographic covariates in prior CEPS-based work, which suggests the main patterns are not entirely driven by the omitted traits flagged earlier (intrinsic motivation, conscientiousness, self-control), though modest inflation from such sources cannot be ruled out.

## 5 Discussion

In this study, the relationships between social support, educational expectations, and normative behavior are examined in the context of the rule of law education of Chinese adolescents. Based on a nationally representative sample drawn from the China Education Panel Survey, the empirical findings reveal patterns consistent with the proposed mediation model. The positive associations of parental, teacher, and peer support with normative behavior appear to be both direct and partially mediated by elevated educational expectations linked to social support. The empirical results of this research offer significant theoretical and practical implications in efforts to interpret the behavioral development of Chinese adolescents and interventions of education operating in the framework of the rule of law education.

The finding that parental support had the strongest direct effect on normative behavior is also supported by the large amount of existing research that emphasizes the central role of family influence during adolescence. As predicted by the parenting for success approach, the findings of this investigation show that parents who provide emotional support, communication, and involvement in education help build an environment within families that encourages behavioral conformity (Fute et al., 2024). The important correlation between parental support and decreased problem behaviors can be understood through the lens of social control theory, whereby strong attachment between parent and child promotes internalization of beliefs about norms. Research examining correlations between perceived parental expectation and engagement in academic activities also suggests that parental engagement communicates both belief and behavioral norms that influence youth behavior (Wang and Tambi, 2024). The present findings extend this literature by demonstrating that parental support influences not only academic outcomes but also broader patterns of normative behavior relevant to rule of law education objectives.

Teacher support was found to be a key predictor for both expectations and normative behaviors, with strong multi-group effects for rural youngsters. The compensatory role played by teacher support is consistent with literature that identifies teachers as important agents of socialization who have the potential to mitigate family resource effects during adolescence (Hu and Qian, 2025). This finding that the effect of teacher support on the educational expectations for rural students is significantly stronger than that for urban students (β = 0.24 vs. 0.15) means that teachers take on a central role in such settings, especially when the role of the parent's educational resource is limited. Recently, the role that academic support by teachers plays in lessening externalizing problems among Chinese adolescents has been consistently documented (Shao et al., 2025). Strategies of classroom management that strengthen a sense of connection between students and their schools have been found particularly effective at encouraging normative behavior among disadvantaged students (Wilkins et al., 2023). This study confirms that teacher support is an equalizing factor that can bring positive behavioral outcomes to students from varied levels of Chinese socioeconomics.

The institutional setting of rural China provides a distinctive context that helps explain this compensatory mechanism (All-China Women's Federation, 2013; Xu and Fu, 2024). Rural labor migration remains a widespread phenomenon in Chinese society, with roughly 61 million children classified as “left-behind children” (liushou ertong) whose parents work in urban centers, which in turn reduces parental academic socialization at home (All-China Women's Federation 2013; Xu and Fu, 2024). Rural schools also lack the auxiliary resources and tutoring programs available to their urban counterparts, so classroom teachers often become the main institutional source of motivation during the pathway to college admission (Rakesh et al., 2025). Teachers in rural communities typically occupy a relatively high social status, and their expectations carry greater motivational weight among rural adolescents than those of other adults (Shao et al., 2025). Against this backdrop, teacher-student relationships function as a substitute for the family academic capital that many rural households lack (Rakesh et al., 2025; Xu and Fu, 2024).

While the part of peer support played in facilitating and promoting normative behavior was statistically significant, its magnitude was relatively modest. This result is consistent with the findings that peer influences toward the behavior of adolescents occur through multiple processes involving socialization, moral pressure, and emotional support (Brass et al., 2024). Research on peer attachment and prosocial behavior suggests that peer relationships play their primary role in behavioral adjustment with respect to empathy and social competence development rather than regulating behaviors directly (Schoeps et al., 2020). In the rule of law education environment, peer support seems to play its role in influencing class environments that promote institutional norms and expectations rather than through processes involving dyadic relationships. The result that peer support effects are stable across gender and residence categories suggests that peer effects for normative behavior are mechanisms with high universality and are not bound by social demographics.

The mediating analysis shows that expectations with respect to educational attainments work as an important cognitive-motivational mediator between social support and normative behavior. This finding supports the predictions of expectancy-value theory, suggesting that an individual's behavioral decisions are influenced by their hope for success and the value placed on achieving particular goals (Eccles and Wigfield, 2020). Adolescents who hold elevated educational expectations recognize that norm-violating behaviors jeopardize their academic futures and develop motivations for protecting their goals through behavioral congruence. Similar chain-mediation effects for the relationship between social support and educational achievement have been uncovered in studies (Chen et al., 2023). The present findings extend this framework by demonstrating that educational expectations mediate not only academic performance but also broader patterns of normative behavior consistent with citizenship education objectives.

This indication of partial mediation rather than complete mediation suggests that there are a variety of pathways leading from social support to normative behavior. The fact that direct effects remained significant after adjusting for expectations related to education suggests additional pathways that may include processes such as emotional control or direct communication of normative values and attitudes (Li et al., 2025). Studies on teacher autonomy support show that supporting teacher-student relationship influences the emotional experiences of adolescents, and their behavioral styles, in ways that are unrelated to achievement beliefs (Çelik, 2024). A comprehensive model for understanding the varied pathways of influence can be gained by integrating the principles of social control theory with expectancy-value theory, where social support enhances the social ties that inhibit deviant actions, and the belief systems that enable supportive actions.

Although the present results show that higher expectations predict more normative behavior, a complete account also needs to consider the potential costs of unrealistically high aspirations. In China, the high-stakes gaokao system adds a motivational layer that may shape whether elevated aspirations translate into beneficial or harmful outcomes. Evidence from high-pressure examination systems such as South Korea's “examination hell” shows that sustained achievement pressure is associated with exam anxiety, sleep deprivation, and emotional exhaustion among adolescents (Lee and Larson, 2000; Steare et al., 2023). Related work has found that parental psychological control, a common correlate of academic pressure, produces maladaptive achievement-goal orientations and undermines autonomous motivation (Xu et al., 2024). Taken together, these findings suggest that expectations may relate to behavioral outcomes in a non-linear way, such that extreme levels impose stress costs that can offset the protective role of moderately high aspirations.

Results from the multiple groups analysis revealed the presence of considerable variability in the structural coefficients for different groups arranged according to their residential status. The significant influential role of parental support for urban students, compared to the role of teacher support for rural students, reflects the unequal allocation of resources within the educational context. Research on parental educational attainment and adolescent development has documented that urban families typically possess greater educational capital that enables more effective academic socialization (Xu and Fu, 2024). In this case, rural school-going children may fall back on school-based support systems more extensively to compensate for a lack of academic resources within the family. These findings are consistent with research findings that highlight the importance of contextual resources and limitations that determine the relative importance of various sources of support within youth development and protective factors (Rakesh et al., 2025). Educational interventions within the rule of law education framework should therefore consider these contextual variations when designing programs to promote normative behavior among diverse student populations.

This absence of stark gender differences in the structural model suggests that the mechanisms by which social support affects normative behavior are similar for both male and female adolescents. This finding contradicts literature that suggests gender differences in the strength of peer influences on behavioral outcomes but supports findings in literature suggesting that family and school support processes are similar across gender groups (Dawes et al., 2024). The continuous mediational role of expectations of educational achievement across genders indicates that the goal-protection process is a universal pathway through which nurturing relations bring about conformity of behavior, irrespective of gender identity.

Another significant issue is the cultural boundaries within which these findings hold. Certain effects may be based on fairly universal dynamics: the effect of social support on reducing adolescents' problem behaviors has already been amply confirmed within various cultural environments (Bauer et al., 2021; Wang et al., 2024), and the expectancy-value model is equally valid for North American, European, and Asian communities (Eccles and Wigfield, 2020). At the same time, some factors may be considered culturally embedded. Specifically, the prominence of parental influence can be explained by Confucian ideas of filiality and achievement-related orientation within families. The importance attached to institutional conformity as an output may be associated with the collectivist normative orientation inherent in fazhi jiaoyu; thus, it might be difficult to apply this concept to cultures where civic norms are determined by claims to rights. Further comparative studies, contrasting results obtained in South Korea, Vietnam, and Japan with Western samples, could reveal which processes transcend culture and which depend on Chinese institutional specifics.

Several limitations should be borne in mind when interpreting these findings. The cross-sectional CEPS design rules out causal inference across social support, educational expectations, and normative behavior, and three sources of potential bias deserve explicit attention. First, reverse causation is plausible, since adolescents who already display normative behavior may elicit more support from parents, teachers, and peers rather than the reverse. Second, omitted variables such as intrinsic motivation, conscientiousness, or self-control may jointly drive help-seeking, educational aspirations, and normative behavior, and could inflate the observed associations even after conditioning on the rich covariate set used here. Third, reliance on student self-report across all constructs raises concerns about common method variance, though Harman's single-factor test yielded a first factor accounting for only 28.6% of the variance, which suggests this is unlikely to be a major source of bias. A further consideration is that the CEPS sample was collected in the 2013–2014 academic year, predating a decade of change in China's educational and social environment, so direct generalizability to current cohorts may be attenuated. The reported estimates should therefore be read as upper-bound associations rather than as causal effects.

Future research should use longitudinal designs that can establish temporal precedence and developmental trajectories between variables of interest, and these should be directed at overcoming the limitations listed above. Using multi-informant designs that combine parent, teacher, and peer ratings will increase the integrity of findings and also permit evaluation of the degree of congruence between individuals' perceptions and other people's perceptions of supports and behavior. Intervention studies, which experimentally manipulate aspects of social support, would provide stronger causal claims and help develop evidence-based interventions for the rule of law education program. Cross-cultural studies would help establish if these factors are identified as unique mechanisms in the context of Chinese education, as compared to their applicability in a generic, universal manner. Studies exploring factors which moderate, such as school environment, neighborhood, and family structure, would help articulate a set of conditions under which support would most effectively impact adolescents.

## 6 Conclusion

This study investigated the linkages between social support, educational aspirations, and normative behavior, in the context of rule of law education for Chinese adolescents, based on nationally representative data from the China Education Panel Survey (*N* = 18,426). Results, based on structural equation modeling, indicate that social support by parents (β = 0.24), teachers (β = 0.15), and peers (β = 0.11) was significantly associated with adolescent normative behavior. The mediating effects of educational expectations partially explained these associations, though the indirect effects ranged from 0.025 to 0.058, representing about 18.5% to 19.5% of the total effects. The multi-group analysis revealed significant differences in the strength of associations of teacher support in shaping educational expectations in rural (β = 0.24) and urban students (β = 0.15), which suggests the compensatory function of support in school in low-resourced areas. These findings point to the value of cultivating coordinated support systems across family, school, and peer contexts to promote adolescent normative behavior consistent with rule of law education objectives in China. Given the cross-sectional observational design of the current study, these implications should be interpreted as exploratory rather than prescriptive, and future longitudinal work is needed to empirically adjudicate the directional pathways among social support, educational expectations, and normative behavior.

## Data Availability

Publicly available datasets were analyzed in this study. This data can be found here: China Education Panel Survey (CEPS), https://ceps.ruc.edu.cn/.
